# Dietary trajectories over 21 years and frailty in Norwegian older adults: the Tromsø Study 1994–2016

**DOI:** 10.1007/s00394-024-03482-z

**Published:** 2024-08-28

**Authors:** Dina M. Konglevoll, Lene F. Andersen, Magne Thoresen, Torunn H. Totland, Laila A. Hopstock, Anette Hjartåker, Monica H. Carlsen

**Affiliations:** 1 Department of Nutrition, Institute of Basic Medical Sciences, University of Oslo, Oslo, Norway; 2 Department of Biostatistics, Institute of Basic Medical Sciences, University of Oslo, Oslo, Norway; 3 Department of Physical Health and Ageing, Norwegian Institute of Public Health, Oslo, Norway; 4 Department of Health and Care Sciences, UiT The Arctic University of Norway, Tromsø, Norway

**Keywords:** Dietary trajectories, Frailty, Frailty index, Nordic nutrition recommendations, NNR 2023, Older adults

## Abstract

**Purpose:**

To investigate the association between five dietary trajectories over 21 years and frailty in Norwegian older adults.

**Methods:**

This study used data from three surveys of the Tromsø Study. Diet was measured using food frequency questionnaires at baseline (Tromsø4, 1994–95), after 7 years (Tromsø5, 2001) and at the end of follow-up (Tromsø7, 2015–16). Survey-specific diet scores were constructed based on the Nordic Nutrition Recommendations 2023 and group-based trajectory modelling was used to derive dietary trajectories. At follow-up, frailty was assessed with a 41-item frailty index. Linear regression analysis was performed to assess the associations between dietary trajectories and frailty, adjusted for baseline variables.

**Results:**

Among the 715 participants, 55% were women, with an average age of 54 years at baseline and 74 years at follow-up. The dietary trajectories ‘moderately healthy’ and ‘healthy increase’ were associated with a lower frailty index score at follow-up (*β* = −0.02, 95% confidence interval (CI) = −0.04, −0.002, *β* = −0.03, 95% CI = −0.06, −0.007), compared with the ‘unhealthy’ trajectory.

**Conclusion:**

Our findings suggest that maintaining a moderately healthy to very healthy diet from mid-life into older age is associated with a lower risk of frailty and supports the promotion of a healthy diet from adulthood to facilitate healthy ageing.

**Supplementary Information:**

The online version contains supplementary material available at 10.1007/s00394-024-03482-z.

## Introduction

As the world’s population ages, the prevalence of geriatric syndromes, such as frailty, has been increasing [1]. Frailty is a complex syndrome that results from a decline in multiple physiological systems over the course of life [2], leading to increased vulnerability to stressors [2–4]. Despite being associated with increased risk of adverse outcomes and ill-health, frailty has shown to be both reversible and dynamic [5], thus intervention with effective and targeted measures may prevent and/or reverse its development.

The assessment of frailty is mainly done using one of two methods: Rockwood’s frailty index [5] or the physical frailty phenotype [2]. The definition of the frailty index defines frailty by counting the number of health deficits present in an older individual [5], including physical, mental and social health [6, 7]. The physical frailty phenotype focuses on physical characteristics only [2]. Using the frailty index, the sum of the deficits present is divided by the number of deficits counted, resulting in a ‘frailty’ score between 0 and 1.

Diet is a major risk factor for the development of frailty [8–10]. Previous studies on single nutrients and foods have shown that healthy dietary components, such as low-fat dairy, fruit and vegetables and fish, are associated with lower risk of frailty [10–12]. However, as it is the combined content of nutrients and foods of a composite diet that influence health, recent studies have shifted towards investigating the effect of overall diet on health [13, 14] and frailty [15, 16]. The diverse set of nutrients and food components in a composite diet influence many and different aspects of health. Measures of overall diet quality is therefore of importance when elucidating the potential associations between diet and frailty when frailty is measured by the frailty index method, which includes multiple health domains. Most studies on overall diet so far have used the physical frailty phenotype as frailty measure. Two recent systematic reviews found that adherence to the Mediterranean diet [17] and dietary patterns characterized by a high content of fruit, vegetables, whole grains and fish [15] were inversely associated with the risk of developing physical frailty [15, 17]. Moreover, longitudinal studies have shown that higher consumption of healthy plant foods was associated with a lower risk of frailty [18, 19] and accelerated ageing [20], whereas the opposite was seen for diets rich in unhealthy plant foods. Similarly, other longitudinal [21, 22] and cross-sectional [23–25] studies have reported an inverse association between higher diet quality and frailty. Although different diets have been assessed in these studies, they resemble each other in essence because they are characterised by high intakes of vegetables, fruit, whole grains, legumes, healthy fats and oils, moderate intakes of dairy and fish, and low intakes of red and processed meat, unhealthy fats, sweets and snacks – very much in line with the Nordic Nutrient Recommendations (NNR) 2023 [26]. Thus, studies suggest that there seems to be an overall preventive effect on frailty as a result of adhering to current dietary guidelines or complying with healthy dietary patterns.

However, most studies conclude that additional, longitudinal studies are needed to confirm the associations observed between dietary patterns and frailty [15, 17]. Emerging research suggests that changes in diet in adulthood may have consequences for chronic conditions [13] and several health, cardiometabolic measures [27–29], and physical [30] and cognitive function [31, 32] in older age. The evolution of diets or dietary changes over time is known as a dietary trajectory [13] and most studies of dietary trajectories are performed in children, adolescents and early adulthood [33]. To our knowledge, there are no previous studies on trajectories and frailty, although studies have looked at dietary trajectories and frailty-related health outcomes in adults and older adults. For example, Talegawkar et al. showed that improving diet quality in mid-life was associated with better physical function in older age [30]. Studies have also reported that patterns of consistent high or improved dietary quality over time were associated with improved cardiometabolic outcomes [27–29, 34], cognitive health [31, 32], psychosocial well-being [32] and lower mortality rates [19, 35] later in life.

With this in mind, we aimed to investigate the association between trajectories of diet over more than two decades and frailty, assessed using the frailty index, in a sample of middle-aged and older men and women from a Norwegian population-based study.

## Methods

### Study design and population

The Tromsø Study is Norway’s most longstanding, population-based cohort study consisting of seven surveys (Tromsø1–Tromsø7) conducted between 1974 and 2016, including in total 45 473 participants [36, 37]. We included data from Tromsø4 (1994–95, baseline) with 27 158 participants aged 25–97 (72% attendance), Tromsø5 (2001) with 8130 participants aged 30–89 (78% attendance) and Tromsø7 (2015–16, follow-up) with 21 083 participants aged 40–99 (65% attendance) [36, 37]. The follow-up period was 21 years.

Data were collected via questionnaires, biological sampling and clinical examinations (visit 1) [38]. Dietary data were assessed through food frequency questionnaires (FFQs). On attendance, a subsample predefined before the start of the study was invited to participate in additional comprehensive clinical examinations (visit 2) [38].

**Fig. 1:**
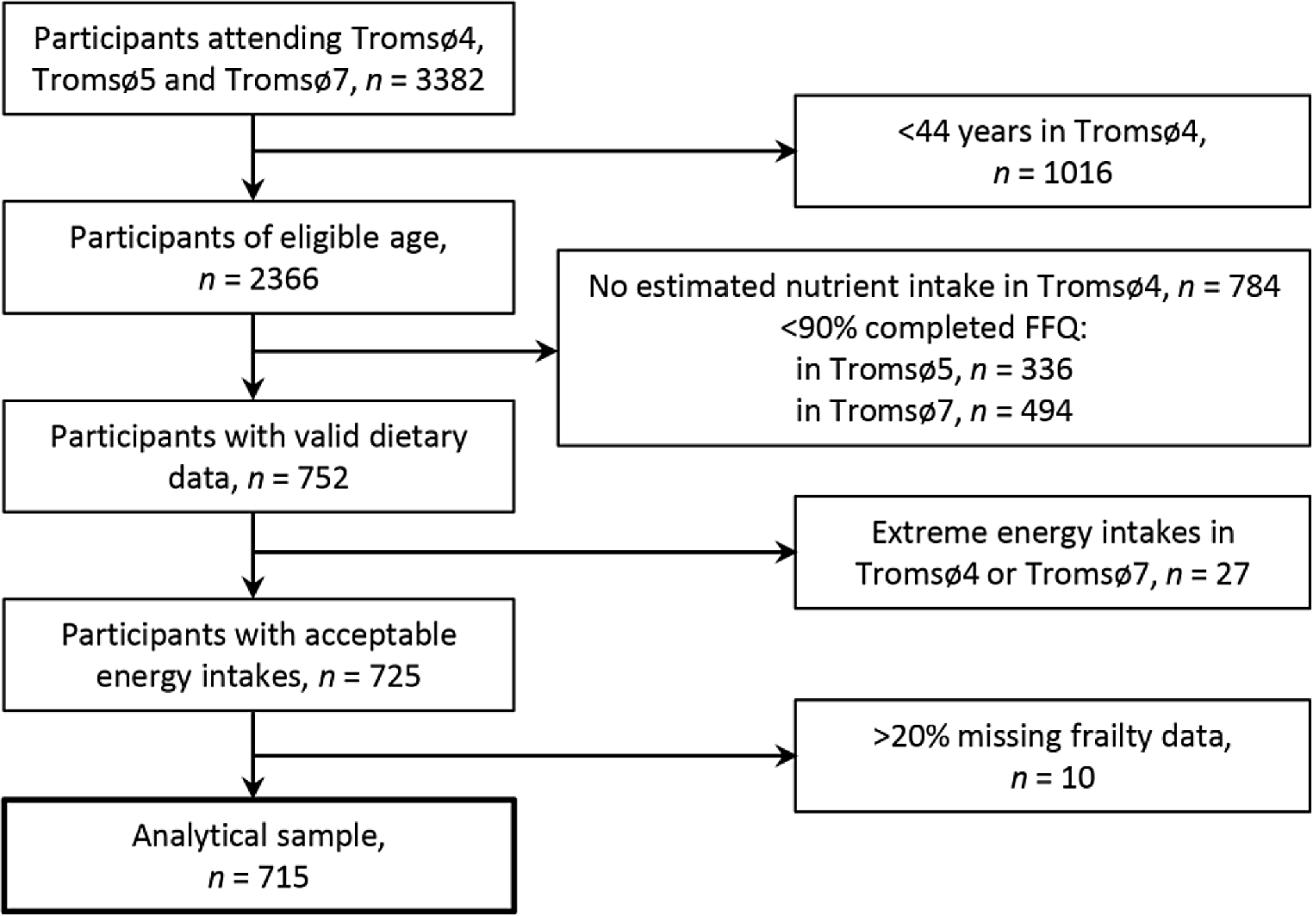
Flow chart of study participants

Our sample included men and women participating in all three surveys (*n* = 3382), who were aged ≥44 years at baseline (i.e. ≥65 years at follow-up, in Tromsø7) (*n* = 2366). We excluded participants without data on estimated nutrient intakes in Tromsø4 (*n* = 784), with < 90% completed FFQs in Tromsø5 (*n* = 336) or Tromsø7 (*n* = 494), or with estimated energy intakes outside < 1st and > 99th percentiles in Tromsø4 and Tromsø7 (*n* = 27), respectively, in accordance with Jacobsen and Nilsen [39] and Lundblad et al. [40]. In addition, we excluded participants with > 20% missing frailty data in Tromsø7 (*n* = 10), leaving 715 participants eligible for the statistical analysis (Fig. 1).

### Dietary assessment

**Fig. 2:**
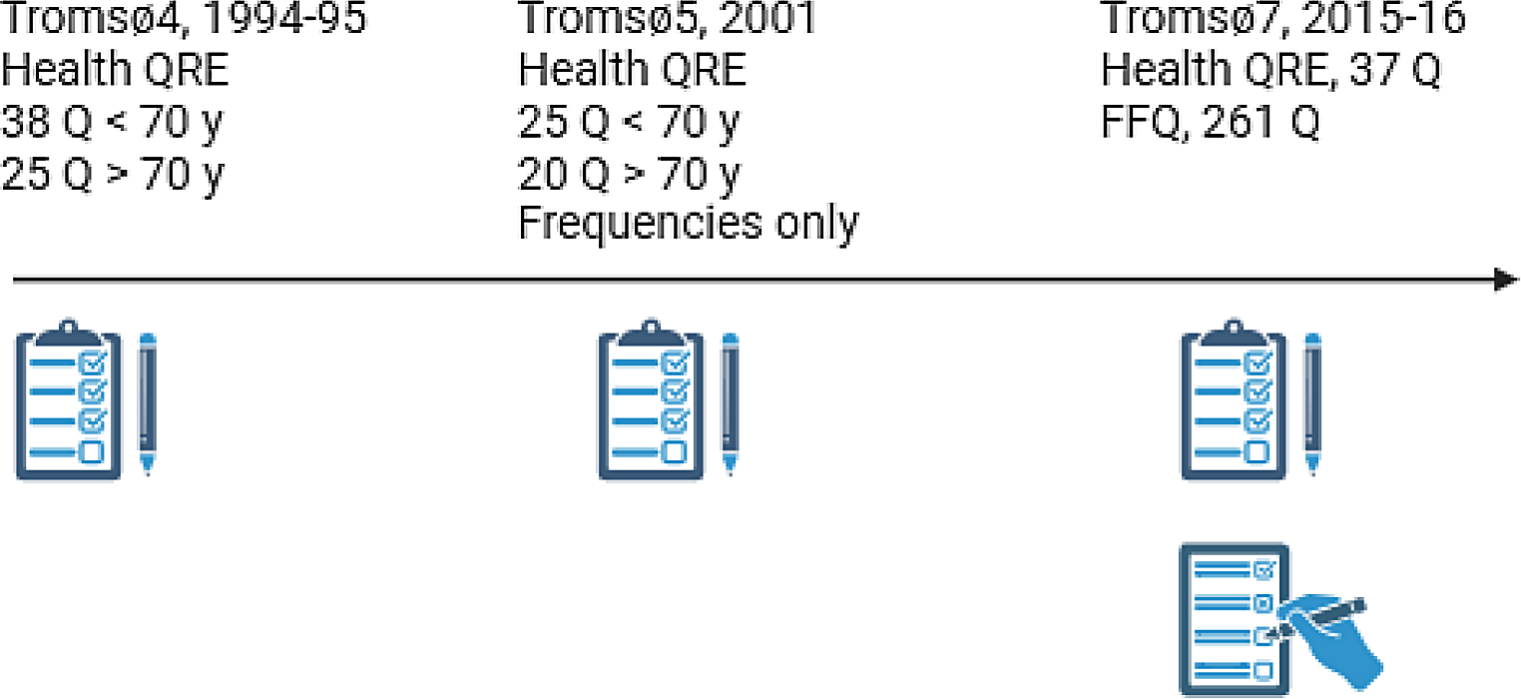
Overview and numbers of diet-related questions in the three included study waves from the Tromsø Study. QRE, questionnaire; Q, questions; y, years; FFQ, food frequency questionnaire

Dietary assessment was self-reported in the Tromsø Study and the methodology has evolved from a few diet-related questions embedded in the overall health questionnaires in the early surveys, to a comprehensive semi-quantitative FFQ in Tromsø7 (2015–16) [40]. An overview of the dietary assessments included in the present study is given in Fig. 2.

The health questionnaires at each survey wave included varying numbers of diet-related questions, see Fig. 2. In Tromsø4 and Tromsø7, questions about both frequency of intake and portions sizes enabled estimation of daily nutrient and food intakes. In Tromsø5 the dietary questions included only questions about frequency of food intake and thus only frequencies could be estimated. Also, in Tromsø4 and Tromsø5 different total numbers of dietary questions were given to participants below and above 70 years of age, see Fig. 2. An overview of the dietary variables from each survey are listed in Supplementary Table S1. In all surveys, dietary data were collected through questions on frequency and preferences of food and drink intake embedded in the health questionnaires; in Tromsø7, a separate semi-quantitative FFQ was also provided. Some foods were consistently asked about in similar ways in all three surveys: coffee and tea, potatoes, fatty fish, milk, fat used in cooking and on bread, alcohol consumption and dietary supplements. Other foods were asked about in only some of the surveys or the wording of the questions differed so much that direct comparisons between surveys were difficult (e.g. asking about frequency of intake of apples/pears and oranges/mandarins in Tromsø4 versus fruit/berries in Tromsø5).

Specifically, dietary data in Tromsø4 were collected via 38 questions on frequency and preferences of food and drink intake. Of these, 34 items were energy-yielding foods that provided the basis for the estimated daily energy and nutrient intake, described in detail by Jacobsen and Nilsen [39]. Nutrient estimations were performed for participants who had answered a minimum of 31 (90%) of the 34 questions, and combined with sex-specific portion sizes, based on data from previous, comparable dietary surveys [41, 42]. Calculations of nutrient intakes were based on the Norwegian (1995) and Swedish (1993) food composition tables [43, 44]. In Tromsø5, 21 frequency and preference questions on intake of common foods and drinks provided the basis for the frequency dietary data. In Tromsø7, in addition to the 37 frequency questions in the health questionnaire, the participants received a separate 261-item semi-quantitative FFQ on different foods, meals and beverages developed by the University of Oslo (UiO) to cover a person’s total diet in the last year [40]. The FFQ provided the basis for the estimation of daily nutrient intakes, the process for which has been described in more detail previously [40]. In brief, daily energy and nutrient intakes were calculated using the food and nutrient calculation system KBS, database version AE14, at the UiO (KBS version 7.3). KBS AE14 is based on the Norwegian food composition table of 2014–15 (https://www.matvaretabellen.no/?language=en) supplemented with data from calculated recipes and other databases [40, 45].

### Diet scores

For each survey, a distinct diet score was created based on the available dietary data, ranging the participants’ diet from least healthy (lowest score) to most healthy (highest score). The scores were constructed based on the recently published NNR 2023 [26] for intake of nutrients and food groups, as an objective marker of a healthy diet. Detailed overviews of the scoring and contents of the diet scores are presented in Supplementary Tables S1 and S2.

Each dietary variable was scored between 0 (least healthy) and 5 (most healthy). Cut-offs for dietary intake were set as in the NNR 2023, supplemented with cut-offs and amounts as in the Norwegian [46, 47] and Danish [48] Dietary Guidelines and Alternative Healthy Eating Index (AHEI) [49]. Where possible, we used estimated daily food and nutrient intakes in the scores, but for a large part – including all Tromsø5 data – we only had data on frequencies of intake. Moreover, when the recommendations in NNR 2023 were open to interpretation, we took cultural considerations into account when quantifying the recommendations [50–54]. For example, we defined ‘a significant part of a healthy diet’ for potatoes as a larger amount (50–200 g or 1–4 potatoes per day) than for pulses (20 g or about 1 tablespoon per day), taking into consideration the potato’s established place in the Norwegian diet compared with the much less commonly eaten pulses [50–52].

Dietary components included in all three scores were coffee, tea, sugar-sweetened drinks, fruit, vegetables, potatoes, juice, fatty fish, low- and full-fat dairy products, alcohol consumption and use of dietary supplements. Similar food groups were scored equally in the three scores and were also weighted equally in each diet score, *relative* to the other dietary variables in that specific score. If the number of sub-variables within food groups differed between surveys, these were scored differently to correct for their varied contribution to the overall score [14]. For example, the food group ‘fish’ was given 5 points in total in all surveys. However, as there was only one question about fish intake in Tromsø5, but four in Tromsø4, the single fish question in Tromsø5 was given a higher score because this alone constituted the fish category, contributing more to the overall score than each of the four questions in Tromsø4 (Supplementary Table S2). The Tromsø4 diet score included a total of 37 dietary variables with a maximum score of 64, the Tromsø5 score; 19 variables with a maximum score of 43 and the Tromsø7 score; 31 variables with a maximum score of 75. For each survey, the participant’s total score was divided by the maximum score, resulting in scores between 0 and 1 for each survey. For example, if a participant in Tromsø4 received a total score of 50, the final, ‘standardised’ diet score would be 50/64 = 0.78.

### Frailty assessment

Frailty was assessed at follow-up only (Tromsø7) using a 41-item deficit accumulation frailty index [5], which included self-reported and objectively measured symptoms, diseases and disabilities. Deficits considered for the frailty index were health and age related, and not too rare (< 1% prevalence) [6]. The included deficits (*n* = 41) covered several health domains: diseases and medication use (*n* = 15), objectively measured physical function (*n* = 6), self-reported health and function (*n* = 8), motivation and attitudes (*n* = 4), vitality and quality of life (*n* = 5) and cognition and memory (*n* = 3) (Supplementary Table S3). For each participant, the sum of all the deficits was divided by the total number of deficits considered, resulting in a frailty index score ranging from 0 (least frail) to 1 (extremely frail). For example, if eight deficits were present, the frailty index score would be 8/41 = 0.20. Participants were categorised as frail using a frailty index score ≥0.25, which is the most commonly used cut-off score in studies of community-dwelling older adults [55]. If data on health deficits were missing, a cutoff of 20% missing deficits were implemented, and the score was calculated based on the specific sum of deficits available for each participant (i.e. minimum 33 and maximum 41 health deficits).

### Covariates

At baseline (Tromsø4), demographic, socioeconomic and health-related information was self-reported via questionnaires. Educational level was categorized as primary (7–10 years), secondary (vocational/middle school, senior high school (1–2 years), high school diploma) and higher (college/university) education. Cohabitation was defined as being married or in a registered partnership and/or living with a spouse or partner. Good self-reported health was defined as answering the two highest answer alternatives (‘Good’, ‘Very good’) to the question ‘What is your current state of health?’. Low physical activity was defined as performing less than 3 h of light exercise (without sweating or being out of breath) per week. Self-reported smoking status was divided into never smoked, previous daily smoker and current daily smoker. Social support was defined as answering ‘Yes’ to the question ‘Do you feel that you have enough good friends?’. Body mass index (BMI) (kg/m^2^) was calculated based on weight (kg) and height (m) measured in light clothing without shoes. Comorbidity was defined as the presence (previous/current) of two or more of the following major non-communicable diseases: self-reported cardiovascular disease (myocardial infarction, angina pectoris, stroke), self-reported chronic lung disease (chronic bronchitis, asthma), self-reported diabetes and/or registered cancer (obtained from the Norwegian Cancer Registry).

### Statistical analysis

Baseline characteristics of study participants were presented as means and standard deviations (SD) for continuous variables, and counts and proportions for categorical variables, combined and stratified by dietary trajectories. Differences between trajectories were assessed using the analysis of variance (ANOVA) for continuous variables and χ^2^ tests for categorical variables. The distribution of continuous variables was assessed visually via histograms and quantile–quantile (Q–Q) plots.

In each survey, the diet scores were divided into quintiles (Q1 to Q5), classifying the participants’ diet as ‘very unhealthy’ (Q1), ‘unhealthy’ (Q2), ‘moderately healthy’ (Q3), ‘healthy’ (Q4) and ‘very healthy’ (Q5). Based on these, we applied group-based trajectory modelling (GBTM) to identify subgroups of participants who followed similar patterns of dietary trajectories over time, using the *traj* command in Stata. The optimal number and shape of the trajectories were determined by comparing Bayesian and Akaike information criterion values of different models [56], resulting in a final model with five distinct trajectories labelled. Naming of the dietary trajectories were based on the observed patterns.

The association between the dietary trajectories and frailty index score was analysed using linear regression models. In all analyses, the ‘unhealthy’ dietary trajectory was the reference group. Two multivariable models were built with variables chosen based on empirical knowledge on the diet–frailty association through careful evaluation of each variable’s contribution to the model and comparisons of versions of models until an optimal fit was found. Model 1 was adjusted for baseline age and sex and model 2 was additionally adjusted for baseline educational level, smoking status, BMI, social support and self-reported health. Educational level was forced into the model despite no statistical influence. No plausible significant interactions were identified, nor were there indications of multicollinearity between the adjustment variables (variation inflation factor < 5).

Variables with > 10% missing were excluded from the construction of the diet scores and the frailty index. In all surveys, missing values for intake of different types of coffee, tea and milk, asked about in the questionnaires, were imputed manually with zero values if the participant had answered some parts of the question. For example, in Tromsø4, if a participant had provided information on daily intake of filtered coffee and tea, but not boiled coffee, we imputed value 0 for boiled coffee. Moreover, as a sensitivity analysis, we applied multiple imputation (MI) on missing food data among the main sample and participants originally excluded owing to unsatisfactory completion (< 90%) of the FFQs, but who had data on ≥25% of the relevant food variables at each time point. Then, 25 imputations were performed with the predictive mean-matching method and estimates were combined using Rubin’s rule [57]. The imputation model included all original food variables that constituted the diet scores, sociodemographic covariates from all surveys, including statistical adjustment variables, and the outcome, the frailty index score in Tromsø7. Five new sample-specific trajectories were identified in the imputed sample with GBTM (Supplementary Fig. S1) and the linear regression analysis was repeated on these trajectories and the frailty index in Tromsø7 (Supplementary Table S4). Comparison of characteristics between the main sample and those additionally included in the MI sample is presented in Supplementary Table S5. Moreover, to address selective drop-out between surveys, we compared characteristics between drop-outs in the main sample and drop-outs after Tromsø4 (Supplementary Table S6).

STATA 17 was used for all statistical analyses. *P* values < 0.05 were considered statistically significant.

## Results

### Dietary trajectories

**Fig. 3:**
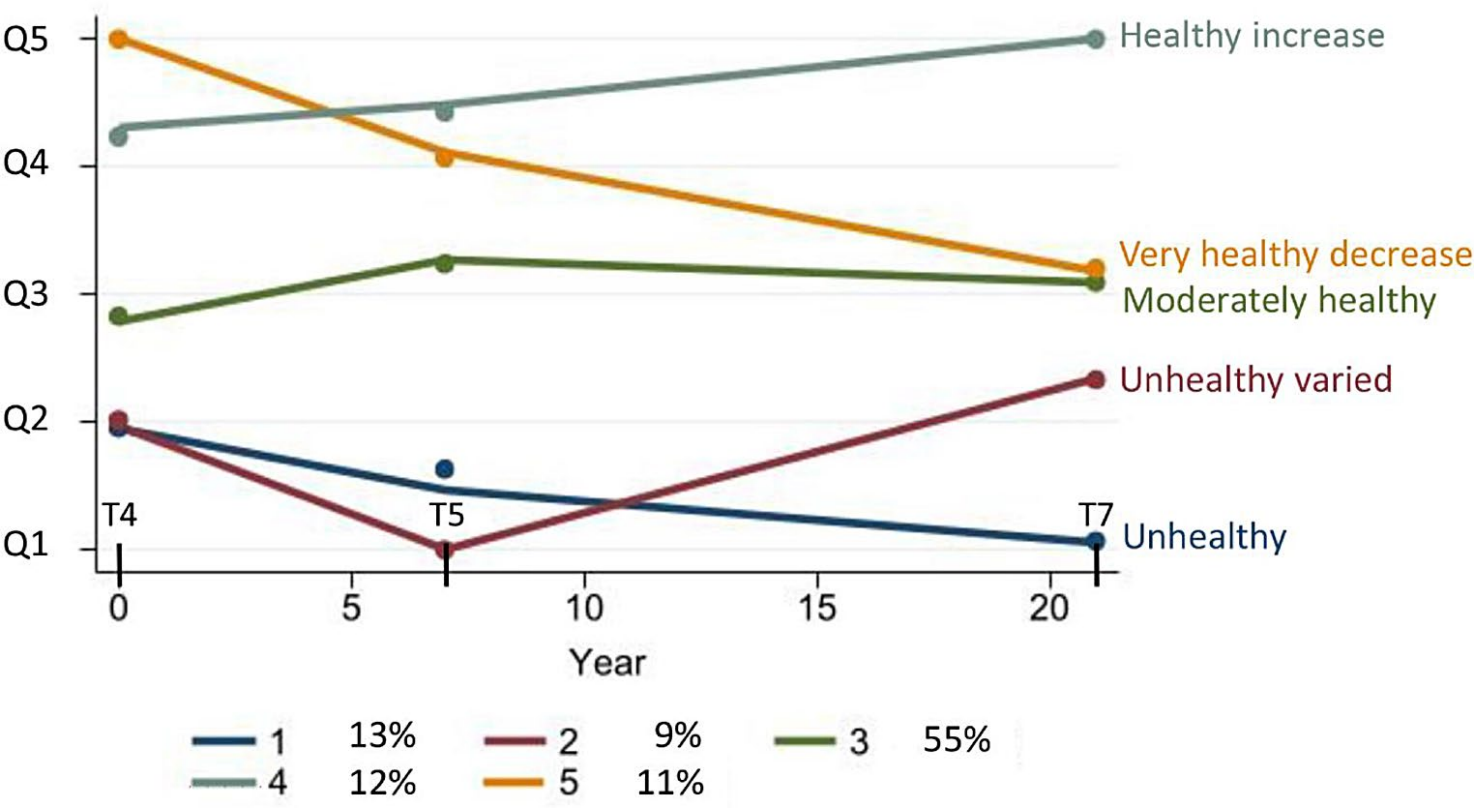
Dietary trajectories: (1) unhealthy, (2) unhealthy varied, (3) moderately healthy, (4) healthy increase, (5) very healthy decrease. Q1–Q5, quintiles of diet scores. T4, Tromsø4 (1994–95), T5, Tromsø5 (2001), T7, Tromsø7 (2015–16)

Based on the quintiles of the three diet scores measured between 1994 and 2016, we identified five dietary trajectory groups (Fig. 3). In total: 13% (*n* = 90) of the participants had a self-reported diet initially classified as unhealthy that gradually decreased to very unhealthy over time (trajectory 1: unhealthy, blue line); 9% (*n* = 63) had a diet first classified as unhealthy, then very unhealthy in Tromsø5 before increasing towards moderately healthy in Tromsø7 (trajectory 2: unhealthy varied, red line); 55% (*n* = 397) of participants had a relatively stable, moderately healthy diet (trajectory 3: moderately healthy, green line); 12% (*n* = 85) had a healthy diet that increased gradually to very healthy over time (trajectory 4: healthy increase, grey line); and 11% (*n* = 80) had an initially very healthy diet that gradually decreased to moderately healthy over time (trajectory 5: very healthy decrease, yellow line).

### Participants’ characteristics and frailty

The participants (55% women) were on average aged 54 years at baseline (74 years at follow-up) with a mean BMI of 25.4 kg/m^2^ (Table 1). The majority cohabited (86%), reported good social support (80%) and considered their own health as good (73%). At baseline, about one-third of the participants were highly educated, one-third daily smokers and one-third physically inactive. Comparison between diet trajectory groups showed that BMI was highest in the ‘unhealthy’ trajectory group. Moreover, participants in the trajectories ‘unhealthy’ and ‘unhealthy varied’ were least likely to be highly educated and most likely to be daily smokers and inactive, whereas the opposite was seen for participants in the ‘healthy increase’ trajectory (Table 1).

At follow-up, the mean frailty index score was 0.22 (range: 0.04–0.54, data not shown) and 31% were classified as frail (Table 1). More women than men were frail and frailty increased with age (data not shown). Comparison between groups showed that frailty was most common in the ‘unhealthy’ trajectory group (38%) and least common in the ‘moderately healthy’ trajectory group (27%).

**Table 1:** Baseline characteristics and follow-up frailty status of participants stratified by dietary trajectories (n = 715)

Baseline characteristics, Tromsø4	Dietary trajectories						
	All (*n* = 715)	Unhealthy (*n* = 90)	Unhealthy varying (*n* = 63)	Moderately healthy (*n* = 397)	Healthy increase (*n* = 85)	Very healthy decrease (*n* = 80)	*P*
Women, *n* (%)	391 (54.7)	39 (43.3)	22 (34.9)	205 (51.6)	65 (76.5)	60 (75.0)	< 0.001
Age (years), mean (SD)	53.9 (5.0)	53.7 (5.2)	53.0 (5.5)	53.7 (5.0)	54.7 (4.7)	54.4 (4.5)	0.26
BMI (kg/m^2^), mean (SD)	25.4 (3.3)	26.3 (4.1)	25.7 (3.3)	25.3 (3.2)	24.8 (3.1)	25.3 (3.1)	0.03
Cohabitant *n* (%)	614 (85.9)	77 (85.6)	53 (84.1)	343 (86.4)	73 (85.9)	68 (85.0)	0.99
Social support^a^, *n* (%)	561 (80.3)	69 (80.2)	53 (85.5)	310 (79.5)	64 (76.2)	65 (85.4)	0.57
Good health, *n* (%)	521 (73.0)	63 (70.0)	49 (77.8)	295 (74.3)	56 (65.9)	58 (73.4)	0.45
Education^b^, *n* (%)							
Primary	211 (29.5)	42 (47.2)	27 (42.9)	110 (27.8)	14 (16.5)	18 (22.5)	< 0.001
Secondary	282 (39.6)	26 (29.2)	22 (34.9)	167 (42.1)	30 (35.3)	37 (46.3)	
Higher	220 (30.9)	21 (23.6)	14 (22.2)	119 (30.1)	41 (48.2)	25 (31.2)	
Smoking, *n* (%)							
Never	254 (35.6)	26 (28.9)	11 (17.5)	141 (35.5)	45 (53.6)	31 (38.8)	< 0.001
Previously	252 (35.3)	25 (27.8)	20 (31.8)	144 (36.3)	27 (32.1)	36 (45.9)	
Daily	208 (29.1)	39 (43.3)	32 (50.8)	112 (28.2)	12 (14.3)	13 (16.3)	
Inactivity, *n* (%)	212 (29.7)	33 (36.7)	22 (34.9)	122 (30.7)	12 (14.3)	23 (29.1)	0.01
Comorbidity^c^, *n* (%)	4 (0.6)	0	0	2 (0.5)	0	2 (2.5)	^d^
**Follow-up**,** Tromsø7**							
Frailty index, mean (SD)	0.22 (0.08)	0.23 (0.09)	0.23 (0.1)	0.22 (0.08)	0.21 (0.08)	0.21 (0.09)	0.34
Frailty^e^, *n* (%)	220 (30.8)	34 (37.8)	23 (36.5)	108 (27.2)	28 (32.9)	26 (32.5)	0.26

*P* values from ANOVA for continuous variables and χ^2^ test for categorical variables

^a^Good social support defined as self-reported satisfied with number of good friends

^b^Education: primary (7–10 years), secondary (vocational/middle school/senior high school/high school diploma), higher education (college/university)

^c^Comorbidity: ≥2 of the following diseases (present or previous): diabetes, cancer, cardiovascular disease, chronic lung disease

^d^No test performed owing to few observations (*n* < 5) in cell

^e^Frailty: ≥0.25

### Dietary trajectories and frailty

**Table 2:** Association between dietary trajectories over 21 years and frailty in Tromsø7 (*n* = 715)^a^

Dietary trajectories	Model 1		Model 2	
	*β*	95% CI	*β*	95% CI
Unhealthy	Ref.		Ref.	
Unhealthy varied	−0.008	−0.04, 0.02	−0.005	−0.03, 0.02
Moderately healthy	−0.03	−0.05, −0.007	−0.02	−0.04, −0.002
Healthy increase	−0.04	−0.06, −0.01	−0.03	−0.06, −0.007
Very healthy decrease	−0.03	−0.06, −0.007	−0.02	−0.05, 0.0008

CI, confidence interval

^a^Linear regression: regression coefficients (*β)* represent the difference in frailty index score at follow-up between the different dietary trajectories, compared with the ‘unhealthy’ trajectory

Model 1: adjusted for baseline age and sex

Model 2: adjusted for baseline age, sex, body mass index, self-reported health, smoking, social support and education

In both simple and fully adjusted analyses, the dietary trajectories ‘moderately healthy’ and ‘healthy increase’ were associated with 0.02 (*β* = −0.02, 95% confidence interval (CI) = −0.04, −0.002) and 0.03 (*β* = −0.03, 95% CI = −0.06, −0.007) lower frailty index score at follow-up compared with the ‘unhealthy’ trajectory (Table 2). In the simple adjusted analysis, the ‘very healthy decrease’ trajectory was also associated with a lower frailty index score; however, this was not statistically significant in the fully adjusted model.

Repeating the linear regression analysis in the MI sample (*n* = 1998, overall 13% missing food data) on sample-specific dietary trajectories (Supplementary Fig. S1 S2) against the frailty index in Tromsø7, compared with the ‘unhealthy’ trajectory, the trajectories ‘moderately healthy’, ‘moderate increase’ and ‘very healthy decrease’, was shown to be associated with lower frailty index in Tromsø7, with estimates similar to the main analysis (Supplementary Table S4). Comparison between the main study sample (*n* = 715) and participants originally excluded owing to missing food data, but included in the MI analysis (*n* = 1283), showed that the main sample had better self-reported health, higher education and were more physically active in Tromsø4, and less frail in Tromsø7 (Supplementary Table S5). Comparison between those who reattended the Tromsø Study after Tromsø4 and those who did not (27%) showed that drop-outs were more likely to be men, younger, more highly educated, daily smokers, physically inactive and unmarried/living alone (Supplementary Table S6).

## Discussion

To our knowledge, this study is the first to investigate the association between dietary trajectories and frailty. In this population-based cohort of 715 middle-aged and older Norwegian adults, we identified five dietary trajectories based on three measures of self-reported diet over 21 years. Our results suggest that maintaining a moderately healthy or very healthy diet through adulthood may be associated with lower frailty in older age.

The observed frailty prevalence (31%) in this otherwise relatively healthy cohort of older adults was higher than the reported global pooled frailty prevalence in studies using the frailty index definition (14%) [58]. In line with the literature, frailty was more common in women and increased with age [5, 58, 59]. Notably, the healthiest dietary trajectory group ‘healthy increase’ constituted the participants with the most favourable health-related characteristics and was associated with lower frailty at follow-up compared with the least healthy trajectory. Similarly, the moderately healthy trajectory was also associated with lower frailty. Although reflecting different levels of healthy diets, because participants following the ‘healthy increase’ trajectory had generally higher diet scores than those in the ‘moderately healthy’ trajectory, both trajectories reflected participants who *maintained* or *improved* the quality of their diet from middle age through older age. Moreover, the results from the MI analysis supported this, because all moderate to healthy dietary trajectories were associated with lower frailty compared with the least healthy diet trajectory.

The observed 0.02–0.03 lower frailty index scores associated with the ‘moderately healthy’ and ‘healthy increase’ diet trajectories, compared with the ‘unhealthy’ diet trajectory, translates to an approximately one-health deficit change in the frailty index (0.025 × 41 = 1). Two longitudinal studies in older adults found that a 0.03 change in frailty index had clinically meaningful implications, defined as a noticeable change in both health or appearance observed by health professionals [60] and health-related quality of life [61]. Moreover, a 0.03 change corresponds to the reported average change in the age-related annual rate of frailty index in community-dwelling older adults [5].

Since, as far as we know, there are no studies on dietary trajectories and frailty, direct comparison with other studies is difficult. Due to the absence of prior research on dietary trajectories and frailty, it is challenging to draw comparisons with other studies, and comparisons are therefore restricted to investigations of dietary trajectories and health-related outcomes, mortality, as well as studies on diet assessed at a single time point and frailty in older individuals. Furthermore, the introduction of younger study cohorts, differing follow-up durations, and distinct dietary trajectories specific to each study further complicates the ability to make comparisons. Nevertheless, several studies have reported findings in line with the present study, showing a beneficial association between improved or consistently healthy dietary habits in adulthood and different health outcomes – all covered in the frailty index. Results from the Baltimore Longitudinal Study of Ageing, which followed participants’ adherence to the AHEI from age 30 years to age 59 years, showed that participants with a ‘greatly improved’ dietary trajectory had better physical function in older age than participants with a ‘moderately improved’ trajectory [30]. Another study that measured adherence to the Dietary Approaches to Stop Hypertension (DASH) diet in an older cohort (≥60 years at baseline) over 23 years reported that participants in the ‘consistently high’ group had a lower risk of poor cognitive, psychological and social health compared with the ‘consistently low’ group [32]. Similarly, dietary trajectories reflecting adherence to the modified AHEI over 6 years were identified in Chinese older adults (≥65 years at baseline) and showed that participants with a stable high-diet quality over time had better cognitive performance than those with improved or deteriorating diet quality [31].

Moreover, studies have assessed the association between dietary trajectories and cardiometabolic outcomes. McNaughton et al. reported that adult British women who, over 17 years, adhered to a dietary pattern characterised by high intake of fruit, vegetables and dairy had lower waist circumference, BMI and blood pressure in older age [28]. Xu et al. found an association between adhering to a ‘traditional’ Chinese diet characterised by rice, pork and vegetables, and having a decreased BMI, weight and waist circumference after 7 years, in adults aged ≥60 years [27]. Batis et al. showed that adults with a healthier diet quality over 15 years had lower glycated haemoglobin than those with declining or a consistently unhealthy diet [29]. Similarly, Guo et al. found that, in Chinese adults, changing the diet from a relatively low fat/high carbohydrate percentage of total energy intake (E%) to a high fat/low carbohydrate E% over 20 years was significantly associated with obesity, diabetes and mortality [34]. This is in line with the NNR 2023, recommending a diet with 45–60 E% from carbohydrates and 25–40 E% from fat [26].

These reports of beneficial health effects in older age from adhering to various definitions of healthy diets are supported by longitudinal studies on dietary patterns measured at a single point in time, showing that, overall, adhering to healthier diets is associated with lower frailty [62–64], slower frailty progression [62] and a higher likelihood of study-specific definitions of healthier ageing [65, 66] in older adults.

Although the above-mentioned studies have investigated different dietary patterns, they are mostly in essence in line with NNR 2023’s definition of a healthy diet, with emphasis on balance, variation, high intake of plant foods, whole grain, lean dairy and fish, and lower intakes of high sugary foods and red meat [26].

### Methodological considerations

The main strengths of the present study are its longitudinal design and utilization of repeated measures of diet. Moreover, the frailty index fulfilled the requirements of Searle et al. for construction of a robust index [6] handled as a continuous variable, which according to two systematic reviews is a superior measure of frailty that best captures the multidimensionality of the syndrome [67].

A major limitation of this study is its heavy reliance on self-reported data, which is prone to information bias [68]. Furthermore, the diet scores are based on FFQs that differ considerably between surveys, and no validation studies on the dietary data obtained from Tromsø4 or Tromsø5 are available. However, the estimated energy percentage from the macronutrients in Tromsø4 were comparable to that of the first two Norkost surveys in adults (1993–94, 1997), intended to be representative of the Norwegian population aged 16–79 years [39, 50]. The FFQ used in Tromsø7 has been validated in adults and considered appropriate for assessment of the total diet in large population surveys [69–71].

Although the diet scores were constructed based on the same recommendations, the available dietary data and, consequently, the interpretation of the distinct dietary components included in the scores varied greatly. Especially in Tromsø5, available dietary data were insufficient and did not reflect a complete diet. Despite our efforts to interpret the NNR 2023 and to score the dietary variables equally at each time point, and to average the scores so that all ranged between 0 and 1, they are not directly comparable. We cannot specify exactly what the participants in the different dietary trajectories have eaten, or provide an objective measure of an optimal, long-term diet for the prevention of frailty in Norwegian older adults. Hence, we stress that the identified trajectories measure a *relative* healthy diet, as in a *healthier diet* relative to a *less healthy diet* over time, based on varying self-reported data measured three times over 21 years.

Of note, the framework of the diet scores, the NNR 2023, did not target the specific study population and was published after the Tromsø surveys had been conducted. The NNR 2023’s target group is the general population, covering all ages and individuals with and without chronic diseases [26]. Moreover, as health and diet trends, beliefs and recommendations change with time, one might question the suitability of using dietary recommendations from 2023 to assess diets measured in 1994, 2001 and 2015. However, we chose NNR 2023 because it is the most up-to-date dietary guideline covering all nutrients and common food groups, enabling an objective assessment of a larger proportion of dietary variables in the Tromsø Study than other existing dietary guidelines or diet scores, considering the limited available dietary data in the Tromsø surveys, apart from Tromsø7. Moreover, we did not measure the participants’ *adherence to dietary guidelines*, but attempted to measure an objectively healthy diet as defined by current dietary guidelines, based on the available data.

Another major limitation of the study is the single measure of frailty. Frail individuals were not excluded at baseline or in Tromsø5 owing to lack of data and, thus, the study suffers from the risk of reverse causality. Consequently, participants could have developed frailty earlier than in Tromsø7, which could have influenced the estimates. In addition, this study suffered from missing data, which was handled with simple (zero) imputation and MI. The zero imputation was applied only when the questions were partially answered and, thus, we considered the likelihood that the participants’ non-response was not the result of a reluctance to answer the specific question, but an actual null value. For the other missing food variables, we applied the more flexible and complex method of MI [72, 73]. Reassuringly, the results from the MI analysis supported our main findings, adding robustness to our conclusion.

Another limitation is that this study suffers from risk of selection bias – a common limitation in cohort studies – because participants tend to be healthier and have better socioeconomic status than non-attenders [74, 75]. Moreover, the study suffers from attrition bias caused by selective drop-out between surveys, reflected by the notably less favourable characteristics of those who dropped out after participating in Tromsø4 than the re-attenders. Thus, the study sample is small and most probably does not fully represent the general adult Tromsø population. Given these limitations, our results should be interpreted with caution and their generalisation confined to community-dwelling older Norwegian adults of relatively good health.

## Conclusion

Maintaining a moderately healthy to very healthy diet through adulthood was significantly associated with a lower risk of frailty at older age. These findings support promotion of a healthy diet in mid-life, or even earlier, for improved health later in life. However, more studies are needed to confirm the association between long-term dietary habits and frailty.

## Electronic supplementary material

Below is the link to the electronic supplementary material.

Supplementary Material 1

## Data Availability

The legal restriction on data availability is set by the Tromsø Study Data and Publication Committee to control for data sharing, including publication of datasets with the potential of reverse identification of de-identified sensitive participant information. The data that support the findings of this study are available from the Tromsø Study but restrictions apply to their availability, which were used under licence for the present study and so are not publicly available. Data are available from the Tromsø Study Data on application. Contact information: The Tromsø Study, Department of Community Medicine, Faculty of Health Sciences, UiT The Arctic University of Norway; e-mail: tromsous@uit.no. A detailed overview of the data collection process and links to the main questionnaires can be found on the Tromsø Study’s website (https://uit.no/research/tromsostudy). All variables collected in the Tromsø Study can be found online at https://helsedata.no/en/variables/?datakilde=K_TR&page=search.
