# Supplementary material for: Dietary trajectories over 21 years and frailty in Norwegian older adults: the Tromsø Study 1994–2016
Source: Eur J Nutr. 2024 Aug 28;63(8):2987–98. doi: 10.1007/s00394-024-03482-z (PMC11519092; doi:10.1007/s00394-024-03482-z)
Supplement: Supplementary file 1 — Supplementary Material 1 [file 394_2024_3482_MOESM1_ESM.pdf]

Dietary trajectories over 21 years and frailty in Norwegian older adults: the Tromsø Study 1994–2016.

European Journal of Nutrition

Dina M. Konglevoll*, Lene F. Andersen, Magne Thoresen, Torunn H. Totland, Laila A. Hopstock, Anette Hjartåker, Monica H. Carlsen

*Correspondence: Dina M Konglevoll, Department of Nutrition, Institute of Basic Medical Sciences, University of Oslo, Oslo, Norway. d.m.konglevoll@medisin.uio.no

Supplementary Table S1 Recommended intake of dietary items, and accompanying available variables in Tromsø4, Tromsø5 and Tromsø7

			**Dietary variables in the Tromsø Study**		
**Food item**	**Nordic Nutrient Recommendations (NNR) 2023**	**Quantified recommendations**	**Tromsø4 (1994–5)**	**Tromsø5 (2001)**	**Tromsø7 (2015–16)**
**NUTRIENTS**					
**Carbohydrate**	Recommended range	45–60 E%	Carbohydrate (g/day)[a]		Carbohydrate E%
Added sugars	Preferably lower intake	<10 E%	Added sugars (g/day)		Added sugars E%
Dietary fibre	Recommended intake	≥3 g/MJ per day	Fibre (g/day)		Fibre E%
**Protein**	Recommended range	<65 years: 10–20 E%, ≥65 years: 15–20 E%	Protein (g/day)[a]		Protein E%
**Fat**	Recommended range	25–40 E%	Fat (g/day)[a]		Fat E%
PUFA<br>MUFA<br>SFA	Unsaturated fatty acids are preferable over saturated fatty acids	5–10 E%<br>10–20 E%<br><10 E%	PUFA (g/day)[a]<br>MUFA (g/day)[a]<br>SFA (g/day) [a]		PUFA E%<br>MUFA E%<br>SFA E%
TFA	As low as possible	<0.5 E%[b]			TFA E%
	Choose products rich in unsaturated fat (vegetable oils) over saturated fat (butter and tropical oil)			Type of fat used in cooking<br>Type of fat used on bread	
**Sodium**	Recommended range[c]	1.5–2.3 g/day			Sodium (g/day)
**FOODS AND DRINKS**					
**Water**	Preferred choice of drink	≥25% of total drinks[d]		Water (glasses/day)	Water (tap, bottled), milk, drinking yoghurt, Biola/Cultura, juice, nectar, squash, soft drinks and ice-tea with/without added sugar,

					alcoholic beverages, coffee drinks, tea, hot chocolate (g/day)
**Filtered coffee and tea**	Moderate consumption part of a healthy diet	1–4 cups/day	Coffee other than boiled coffee[e], tea (cups/day)	Filtered coffee, tea (cups/day)	Filtered coffee, tea (cups/day)
**Boiled coffee**	As low as possible	<1 cup/day[f]	Boiled coffee (cups/day)	Boiled coffee (cups/day)	Boiled coffee (cups/day)
**Sugar-sweetened beverages[g]**	As low as possible	≤20 g/day[h]	Sugar-sweetened beverages (frequency)	Soft drinks (frequency)[i]	Sugar-sweetened beverages, squash, iced tea, hot chocolate (g/day)
**Whole grain**	Likely benefits of higher intakes	≥90 g/day	Preferred type of bread		Bread wholegrain (50%, 50–100%), crisp bread wholegrain, oatmeal/4grain, muesli unsweetened (g/day)
**Vegetables, fruit and berries**	Diet should constitute a variety of different types of vegetables and fruit	500 to ≥800 g/day[j]			
**Fruit and berries**	Fruit/berries should constitute about half of total vegetable and fruit intake		Apples/pears, oranges/mandarins (frequency)	Fruit and berries (frequency)	Apple, peach/nectarine, pear, banana, orange, grapefruit, melon, clementine, kiwi, grape, berries (strawberry, raspberry, blueberry, cloudberry), dried fruit, raisins, fruit and nut mix (25% fruit), fruit on bread (spread), hermetic fruit (55%), fruit salad (g/day)[k]
**Vegetables**	Vegetables should constitute about half of total vegetable and fruit intake		Vegetables for dinner, carrots, broccoli/cauliflower/cabbage (frequency)	Boiled vegetables, fresh vegetables/ salads (frequency)	Vegetables (minus beans/lentils) (g/day)

**Potatoes**	Boiled/baked potatoes prepared with little fat and salt should be a significant part of a healthy diet	50–200 g/day, 1–4 potatoes/day[l]	Potatoes (frequency)[m]	Potatoes (frequency)[m]	Potatoes boiled/baked (g/day)
**Fruit juice**	Low to moderate intake.	≤1 glass/day[k]	Orange juice (frequency)	Juice (frequency)	Juice (orange, apple/other) (g/day)[k]
**Pulses/legumes**	Should be a significant part of a healthy diet	≥20 g/day[n], ≥1 tbsp			Beans/lentils (g/day)
**Nuts**	Recommended range	20–30 g/day			Peanuts/cashew nuts, hazelnuts/almonds/walnuts, fruit- and nut mix (65% nuts) (g/day)
**Fish and seafood**	Recommended range	300–450 g/week, about 2–3 dinners	Lean fish for dinner, processed fish for dinner, fish spread on bread (frequency)		Fish/shellfish (g/day)
**Fatty fish**	Recommended intake (of total fish intake)	≥200 g	Fatty fish for dinner (frequency)	Fatty fish (frequency)	Smoked mackerel, salmon, trout, fish spread (smoked salmon/ trout, mackerel in tomato sauce (50% fish), sardines/pickled herring/anchovies) (g/day)
**Red meat (processed and unprocessed)**	Preferably low	≤350 g/week			Unprocessed red meat (roast, steak and chops of pork/beef/lamb, stew with meat), dishes with unprocessed meat (stew with meat [25–50% meat]), processed red meat (sausages of pork/beef [light/not light], hot dog pork/beef, meat

					burger [*karbonade*], meat burger minced meat/meatloaf [60% meat], bacon [96% meat], salami), red meat as spread (boiled ham, bologna [60% meat]), dishes with processed meat (stew with minced meat, tortilla/taco shell with meat (20-60% meat)) (g/day)
**Processed red meat**	As low as possible	≤20 g/day[h]	Processed meat for dinner, meat spread on bread (fatty, lean, processed) (frequency)		Processed red meat, red meat as spread, dishes with processed meat (listed in the above row)
**Low-fat dairy products[o]**	Recommended range	350–500 g/day	Milk (skimmed 0.1%, semi-skimmed 1.2%), yoghurt (frequency)	Milk (skimmed 0.1%, semi-skimmed 1.2%, extra semi-skimmed 0.5%), yoghurt (frequency)	Milk (skimmed 0.1%, semi-skimmed 1.2%, extra semi-skimmed 0.5%), Biola/Cultura: natural, flavoured, yoghurt: drink, natural, fruit, light fruit, cheese light, cream cheese light, cottage cheese, sour cream extra light (10%) (g/day)
**Full-fat dairy products[o]**	Replace whole-fat dairy products with lean dairy products	≤20 g/day[h]	Full-fat milk (3.5%), yellow cheese spread on bread, brown cheese spread on bread (frequency)	Full-fat milk (3.5%), cheese, all types (frequency)	Full-fat milk (3.5%)/Kefir, sour cream (full fat [35%] and light [17%]), cheese (blue, yellow, cream, brown) (g/day)
**Eggs**	Moderate intake may be part of a healthy diet	≤60 g/day, ≤1 egg/day	Eggs (frequency)		Eggs boiled/fried/scrambled (g/day)

**Sweets and sugary foods**	As low as possible	≤20 g/day[h]	Sweet spread on bread, chocolate, waffles/cakes (frequency)		Cakes, buns, waffles, sweet biscuits (bun/roll/pretzel, Danish pastry, muffin, cake, waffle, lefse, chocolate cake, sponge cake, biscuit, "*kokosbolle*", sweet cereals), desserts (ice cream, ice-lolly sorbet, pudding, vanilla sauce, whipped cream), chocolate, candy (milk/dark/confections chocolate, mixed candy, pastilles), salt snacks (crisps, pretzels), sugar-rich spreads (chocolate spread, peanut butter, jam, honey) (g/day)
**Alcohol**	Avoid alcohol intake<br>If consumed, as low as possible		Alcohol (g/day)	Frequency of drinking previous year, units when drinking, frequency of drinking 5 units at once previous year	Alcohol (g/day)
**Dietary supplements**<br>Vitamin D<br>Fish oils<br>Cod liver oil	Regular use is positive[p]		Vitamin D,<br>cod liver oil/fish oil capsules	Vitamin D,<br>cod liver oil	Vitamin D, cod liver oil/cod liver oil capsules, omega-3 capsules (fish oil, seal oil)

E%, energy percentage; MUFA, monounsaturated fatty acids; PUFA, polyunsaturated fatty acids; SFA, saturated fatty acids; TFA, *trans*-fatty acids.
[a]Macronutrients in Tromsø4 not originally available in E%, but calculated to E% via estimated daily energy intake (kcal/day).
[b]Cut-off similarly as in AHEI (Alternative Healthy Eating Index) (44).
[c]NNR2023: adequate intake (i.e. recommended average daily intake expected to meet/exceed the needs of most individuals in a group): 1.5 g/day (3.75 g of salt). Chronic disease risk reduction (i.e. the level above which intake reduction is expected to reduce chronic disease risk): 2.3 g/day (5.75 g of salt) [58].
[d]NNR provides no specific recommendations for quantities of *drinking water*, as the recommended intake of 2–2.5 litres include water from both food and drink. As the water content in food differs greatly, it is difficult to quantify the optimal intake of drinking water specifically. Thus, we chose the cut-off at >25% of total drinks as according to Henriksen et al. [42].
[e]In 1994, we assume all other coffee than boiled coffee was filtered coffee.

[f]Self-defined cut-off. All intakes considered unhealthy.
[g]Does not include artificially sweetened drinks, because NNR2023 does not specifically cover the health effects of their consumption.
[h]Acceptable intake set to one weekly portion (20 g/day) according to Henriksen et al. [42].
[i]Merged variable for participants aged <70 and >70 years, who completed different food frequency questionnaires. Participants aged <70 years were asked about frequency of consumption of cola drinks and non-cola drinks, whereas participants ≥70 years were asked about the frequency of consumption of soft drinks (all types) and mineral water.
[j]Potatoes/pulses not included.
[k]One glass defined as 2 dl (200 g) of juice.
[l]‘A significant amount’ defined as 50–200 g/day, corresponding to about 1-4 potatoes. This is based on the knowledge that potatoes are a staple food in Norway and commonly consumed – especially among the older population [45, 46], and the Danish dietary guidelines recommend a daily intake of about 100 grams [43]. The cut-off was also adapted to reflect the frequency categories in the food frequency questionnaires in Tromsø4 and Tromsø5.
Assume that this is mostly boiled/baked potatoes. Norkost (1993–94) and 2 (1999) found that among Norwegian adults, ‘fresh’ potatoes constituted 95.5% and 93.5% of the total potato intake, respectively. Pommes frites/potato powder constituted the little surplus intake [45].
[n]‘A significant part’ defined as ≥1 tablespoon (about 20 g) per day. This was based on the fact that, until recently, pulses and legumes have not typically been a significant part of the Norwegian diet [45, 46], and in a recent review on global food-based dietary guidelines and consumption of legumes, Norway had the lowest intake of pulses out of 94 countries (about 1.2 g/day) [47].
[o]The classification of ‘lean’ and ‘full-fat’ dairy products is relative and differs between types of dairy products. According to the Norwegian Information Office for Dairy Products, ‘lean dairy products’ are those with lowest fat content in each product group [48]. For example, lean cheese contains up to 17% fat whereas full-fat milk has 3.5% fat. These two products are obviously not comparable in absolute fat content; however, considering that non-lean cheeses contain about 26% fat and the other milk types contain between 0.1% and 1.2% fat, then they are relatively lean and full-fat alternatives, respectively.
[p]NNR 2023 recommends, in general, that a healthy and varied diet shall cover the nutritional needs because there is no scientific reason for using supplements to improve a suboptimal diet, in healthy individuals. However, older adults and individuals with low exposure to sunlight – as inhabitants in Tromsø during the winter months – have a higher risk of low vitamin D intake [49]. Based on this, we considered taking supplements rich in vitamin D (cod liver oil, fish oils, vitamin D) as a positive contribution to the diet in this specific population.

Supplementary Table S2 Scoring of dietary variables in diet scores in Tromsø4, Trosmø5 and Tromsø7

**Tromsø4 (1994–5)**				**Tromsø5 (2001)**				**Tromsø7 (2015–16)**			
**No.**	**Dietary component**	**Max score**	**Score**	**No.**	**Dietary component**	**Max score**	**Score**	**No.**	**Dietary component**	**Max score**	**Score**
	**Carbohydrates**	**5**							**Carbohydrates**	**5**	
1	Total carbohydrate (E%)	**2**	2p = 45–60<br>0p = <45 or >60					1	Total carbohydrate (E%)	**2**	2p = 45–60<br>0p = <45 or >60
2	Added sugars (E%)	**1.5**	1.5p = ≤5<br>1p = 5–10<br>0p = ≥10					2	Added sugars (E%)	**1.5**	1.5p = ≤5<br>1p= 5–10<br>0p = ≥10
3	Dietary fibre (g/MJ per day)	**1.5**	1.5p = ≥3<br>1p = 1.5–3<br>0p = <1.5					3	Dietary fibre (g/MJ per day)	**1.5**	1.5p = ≥3<br>1p = 1.5–3<br>0p = <1.5
4	Protein (E%)	**5**	5p = 10–20<br>0p = <10 or >20					4	Protein (E%)	**5**	5p = 15–20[a]<br>0p = <15 or >20
	**Fat**	**5**			**Fat**	**5**			**Fat**	**5**	
5	Total fat (E%)	**2**	2p = 25–40<br>0p = <25 or >40	1	Fat used in cooking	**2.5**	2.5p = oils<br>2p = do not use<br>1.5p = soft/light margarine<br>1p = hard margarine<br>0p = butter	5	Total fat (E%)	**1**	1p = 25–40<br>0p = <25 or >40
6	PUFA (E%)	**1**	1p = 5–10<br>0p = <5 or >10	2	Fat used on bread	**2.5**	2.5p = oils<br>2p = do not use<br>1.5p = soft/light margarine<br>1p = hard margarine<br>0p = butter	6	PUFA (E%)	**1**	1p = 5–10<br>0p = <5 or >10
7	MUFA (E%)	**1**	1p = 10–20<br>0p = <10 or >20					7	MUFA (E%)	**1**	1p = 10–20<br>0p = <10 or >20
8	SFA (E%)	**1**	1p = <10<br>0p = ≥10					8	SFA (E%)	**1**	1p = <10<br>0p = ≥10
								9	TFA (E%)	**1**	1p = 0<br>0.5p = <0.5

											0p = ≥0.5
								10	Sodium (g/day)	**1**	1p = 1.5–2.0<br>0.5p = 0.5–1.5<br>or 2–2.3<br>0p = <0.5 or ≥2.3
	**Drinks**	**6**			**Drinks**	**8**			**Drinks**	**8**	
9	Tea and<br>filtered coffee<br>(cups/day)	**2**	2p = 1–4<br>0.5p = 0<br>0p = >4	3	Tea and<br>filtered coffee<br>(cups/day)	**2**	2p = 1–4<br>0.5p = 0<br>0p = >4	11	Tea and<br>filtered coffee<br>(cups/day)	**2**	2p = 1–4<br>0.5p = 0<br>0p = >4
10	Boiled coffee	**1**	1p = 0<br>0p = ≥1	4	Boiled coffee<br>(cups/day)	**1**	1p = 0<br>0p = ≥1	12	Boiled coffee<br>(cups/day)	**1**	1p = 0<br>0p = ≥1
11	Sugar-<br>sweetened<br>beverages<br>(per week)	**3**	3p = never<br>2p = <1<br>1.5p = 1<br>1p = 2–3<br>0.5p = ≥4<br>0p = ca. daily	5	Sugar-<br>sweetened<br>beverages	**3**	3p = never<br>1p = 1–6<br>glasses/week<br>0p = ≥1 glasses/day	13	Sugar-<br>sweetened<br>beverages (g/day)	**3**	3p = 0<br>1p = 0–5<br>0.5p = 5–20<br>0p = ≥20
				6	Water<br>(glasses/day)	**2**	2p = ≥4<br>1.5p = 2–3<br>1p = 1<br>0.5p = 1–6<br>glasses/week<br>0p = rarely/never	14	Water<br>(% of total drinks)	**2**	2p = ≥25<br>1p = 12.5–25<br>0.5p = 0–12.5<br>0p = 0
	**Fruit**	**5**			**Fruit**	**5**			**Fruit**	**5**	
12	Oranges,<br>mandarins<br>(/week)	**2.5**	2.5p = daily<br>1.5p = 4–5<br>1p = 2–3<br>0.75p = 1<br>0.5p = <1<br>0p = never	7	Fruit and<br>berries (/day)	**5**	5p = ≥3<br>4p = 1–2<br>2p = 4–6/week<br>1p = 2–3/week<br>0.5p = 1–3/month<br>0p = rarely/never	15	Fruit and<br>berries (g/day)	**5**	5p = ≥250<br>4p = 200–250<br>3p = 150–200<br>2p = 100–150<br>1p = 50–100<br>0.5p = 0–50<br>0p = 0
13	Apples, pears<br>(/week)	**2.5**	2.5p = daily<br>1.5p = 4–5<br>1p = 2–3<br>0.75p = 1<br>0.5p = <1<br>0p = never								

14	Orange juice (glasses/day)	**2**	2p = <1 or 1–2<br>0p = 0 or ≥3	8	Fruit juice	**2**	2p = 1–6 glasses/week or 1 g<br>0p = rarely/never or ≥2 glasses/day	16	Juice (apple, other) (g/day)	**2**	2p = ≤200<br>0p = 0 or >200
	**Vegetables**	**<u>5</u>**			**Vegetables**	**<u>5</u>**			**Vegetables**	**<u>5</u>**	
15	Vegetables (for dinner) (/week)	**2**	2p = daily<br>1.75p = 4–5<br>1.5p = 2–3<br>1p = 1<br>0.5p = <1<br>0p = never	9	Vegetables (boiled)	**2.5**	2.5p = ≥1/day<br>2p = 4–6/week<br>1p = 1–3/week<br>0.5p = 1–3/month<br>0p = rarely/never	17	Vegetables (g/day)	**5**	5p = ≥250<br>4p = 200–250<br>3p = 150–200<br>2p = 100–150<br>1p = 50–100<br>0.5p = 0–50<br>0p = 0
16	Carrots (/week)	**1,5**	1.5p = daily<br>1.25p = 4–5<br>1p = 2–3<br>0.75p = 1<br>0.5p = <1<br>0p = never	10	Vegetables (fresh, salad)	**2.5**	2.5p = ≥1/day<br>2p = 4–6/week<br>1p = 1–3/week<br>0.5p = 1–3/month<br>0p = rarely/never				
17	Cauliflower, cabbage, broccoli (/week)	**1,5**	1.5p = daily<br>1.25p = 4–5<br>1p = 2–3<br>0.75p = 1<br>0.5p = <1<br>0p = never								
18	Potatoes (eaten daily) (/day)	**2**	2p = 1–4<br>0.5p = ≥5<br>0.25p = <1<br>0p = 0	11	Potatoes (times eaten)	**2**	2p = 1–4/day<br>0.5p = 4–6/week<br>0.25p = 1–3/week or 1–3/month<br>0p= rarely/never	18	Potatoes (g/day)	**2**	2p = 50–200<br>0.5p = >200<br>0.25p = <50<br>0p = 0
								19	Legumes (beans/lentils) (g/day)	**4**	4p = ≥20<br>3p = 10–20<br>2p = 5–10<br>1p = 0–5<br>0p = 0
								20	Nuts (g/day)	**4**	4p = 20–30<br>3p = 10–19 or >30

											2p = 5–10<br>1p = 0.1–5<br>0p = 0 g
19	Whole grain (bread type)	**5**	5p = coarse brown<br>2.5p = brown<br>0p = light/white					21	Whole grains (g/day)	**5**	5p = ≥90<br>4p = 70–90<br>3p = 50–70<br>2p = 25–50<br>1p = 0–25<br>0p = 0
	**Fish**	**5**			**Fish**	**5**			**Fish**	**5**	
20	Lean fish (/week)	**1.5**	1.5p = ≥2<br>1p = 1<br>0.5p = <1<br>0p = never					22	Fish and seafood (g/week)	**3**	3p = ≥300<br>2p = 200–300 g/day<br>1p = 100–200<br>0.5p = 0–100<br>0p = 0
21	Fatty fish (/week)	**2**	2p = ≥2<br>1p = 1<br>0.5p = <1<br>0p = never	12	Fatty fish (frequencies)	**5**	5p = ≥4/week<br>4p = 1–3/week<br>1p = 1–3/month<br>0p = rarely/never	23	Fatty fish (g/week)	**2**	2p = ≥200<br>1.5p = 100–200<br>1p = 50–100<br>0.5p = 0–50<br>0p = 0
22	Fish spread (on bread, slices) (/day)	**0.75**	0.75p = ≥1<br>0.5p = <1<br>0p = never								
23	Processed fish (/week)	**0.75**	0.75p = ≥1<br>0.5p = <1<br>0p = never								
	**Meat**	**3**							**Meat**	**3**	
24	Processed meat (g/week)	**1**	1p = never<br>0.5p = <1<br>0p = >1					24	Processed red meat (g/day)	**1**	1p = 0<br>0.66p = <5<br>0.33p = 5–20<br>0p = ≥20
25	Fatty meat spread (on bread, slices)	**1**	1p = never<br>0.5p = <1/week<br>0p = >1/day					25	Red meat (g/week)	**2**	2p = 0<br>1.5p = ≤100<br>1p = 100–250<br>0.5p = 250–350<br>0p = ≥350

26	Lean meat spread (on bread, slices)	**1**	1p = never<br>0.5p = <1/week<br>0p = >1/day								
	**Dairy**	**5**			**Dairy**	**5**			**Dairy**	**5**	
	Low-fat dairy	*3*			*Low-fat dairy*	*3*			*Low-fat dairy*	*3*	
27	Low-fat milk (glasses/day)	**2**	2p = ≥2<br>1p = 1<br>0.5p = <1<br>0p = 0	13	Low-fat milk (glasses/day)	**3**	3p = ≥ 4<br>2p = 2–3<br>1p = 1<br>0.5p = <1<br>0p = 0	26	Low-fat dairy products (g/day)	**3**	3p = ≥350<br>2p = 200–350<br>1p = 50–200<br>0.5p = 0.1–50<br>0p = 0
28	Yoghurt (/week)	**1**	1p = ≥4<br>0.75p = 2–3<br>0.5p = 1<br>0.25p = <1<br>0p = 0								
	Full-fat dairy	*2*			*Full-fat dairy*	*2*			*Full-fat dairy*	*2*	
29	Full-fat milk (glasses/day)	**1**	1p = 0<br>0.5p = <1<br>0p = >1	14	Full-fat milk (glasses/week)	**1**	1p = rarely/never<br>0.5p = 1–6<br>0p = >1 glasses/day	27	Full-fat dairy products (g/day)	**2**	2p = <5<br>1p = 5–20<br>0p = ≥20
30	Yellow cheese (on bread, slices) (/day)	**0.5**	0.5p = <1<br>0.25p = 1–2<br>0p = 0 or ≥3	15	Cheese	**1**	1p = ≤1–3/week<br>0.5p = 4–6/week or 1–2/day<br>0p = rarely/never or ≥3/day				
31	Brown cheese (on bread, slices) (/day)	**0.5**	0.5p = <1<br>0.25p = 1–2<br>0p = 0 or ≥3								
32	Eggs (/week)	**2**	2p = ≥4<br>1.5p = 2–3<br>1p = 1<br>0.5p = <1<br>0p = never					28	Eggs (g/day)	**2**	2p = ≤60<br>0p = >60
	**Sweets and snacks**	**3**							**Sweets and snacks**	**3**	
33	Sweet spread (on bread, slices) (/day)	**1**	1p = 0<br>0.5p = <1<br>0p = >1					29	Sweets and snacks (g/day)	**3**	3p = 0<br>1.5p = 0–5<br>1p = 5–10

											0.5p = 10–20<br>0p = ≥20
34	Chocolate (/week)	**1**	1p = never<br>0.5p = <1<br>0p = >1								
35	Waffles, cakes, etc. (/week)	**1**	1p = never<br>0.5p = <1<br>0p = >1								
	**Alcohol**	**3**			**Alcohol**	**3**			**Alcohol**	**3**	
36	Alcohol (g/day)	**3**	3p = 0<br>1p = ≤5<br>0p = >5	16	Frequency of drinking (/month)	**1**	1p = never/not last year<br>0.5p = ≤1<br>0p = >1	30	Alcohol (g/day)	**3**	3p = 0<br>1p = 0–5<br>0p = >5
				17	Units when drinking (glasses)	**1**	1p = ≤2<br>0.5p = 2–5<br>0p = >5				
				18	5 units on one occasion (times)	**1**	1p = 0/last year<br>0.5 p = 1–5/last year<br>0p = >5/last year				
	**Dietary supplements**	**3**			**Dietary supplements**	**3**			**Dietary supplements**	**3**	
37	Vitamin D, omega-3, cod liver oil	**3**	3p = use regularly<br>1p = use sporadically<br>0p = do not use	19	Vitamin D, cod liver oil	**3**	3p = use regularly<br>1p = use sporadically<br>0p = do not use	31	Vitamin D, omega-3, cod liver oil	**3**	3p = use regularly<br>1p = use sporadically<br>0p = do not use
**37**	**Max. score:**	**64**		**19**	**Max. score:**	**43**		**31**	**Max. score:**	**75**	

E%, percentage energy; MUFA, monounsaturated fatty acids; p, point; PUFA, polyunsaturated fatty acids; SFA, saturated fatty acids; TFA, *trans*-fatty acids.
[a]Categories, frequencies and intervals may differ between surveys for similar food items according to the wording and nature of the original variables in the Tromsø Study dataset.

Supplementary Table S3 Contents and scoring of frailty index in Tromsø7

	**Health deficits within main categories**	**Scoring**	
**No.**	**Diseases and medication use**		
1	Diabetes	Yes = 1, Previously = 0.5, No = 0	
2	Cancer	Yes, now or previously = 1, No = 0	
3	Stroke	Yes, previously = 1, No = 0	
4	Cardiovascular disease	Yes = 1, Previously = 0.5, No = 0	
5	Pulmonary disease	Yes = 1, Previously = 0.5, No = 0	
6	Inflammatory disease	Yes = 1, Previously = 0.5, No = 0	
7	Incontinence	All the time =1, Several times a day = 0.8, Once a day = 0.6, 2–3 times a week = 0.4, Once a week or less often = 0.2, Never = 0	
8	Indigestion/abdominal pain	Yes = 1, Previously = 0.5, No = 0	
9	Severe/chronic pain	Severe = 1, Moderate = 0.5, No = 0	
10	Thyroid hormone medicines	Yes = 1, Previously = 0.5, No = 0	
11	Hearing impairment	Yes = 1, No = 0	
12	Other disease	Yes = 1, Previously = 0.5, No = 0	
13	Psychological problems	Yes = 1, Previously = 0.5, No = 0	
14	Polypharmacy (≥5 medications daily)	Use ≥5 medications daily = 1, Use <5 medications daily = 0	
15	Low haemoglobin levels	<12 g/dl (women), <13 g/dl (men) = 1, ≥12/13 g/dl = 0	
	**Objectively measured physical function**		
16	SPPB: balance test (s)<br>*Feet-gathered posture*<br>*Semi-tandem posture*<br>*Tandem posture*	 <10 s = 0.25, ≥10 s = 0<br><10 s = 0.25, ≥10 s = 0<br><3 s = 0.5, 3–9.9 s = 0.25, ≥10 s = 0	
17	SPPB: walking speed (s/m)	Not able to perform test per >8.7 s = 1, 6.21–8.70 s = 0.75, 4.82–6.20 s = 0.5, <4.82 s = 0	
18	SPPB: chair stand test (s)	Not able to perform per >60 s = 1, ≥16.7 s = 0.75, 13.7–16.69 s = 0.5, 11.20–13.69 s = 0.25, ≤11.19 s = 0	
19	Grip strength (kg)	*Men*<br>BMI ≤24 and GS ≤29<br>BMI 24.1–28 and GS ≤30<br>BMI >28 and GS ≤32 = 1<br><br>GS above cut-off within BMI strata = 0	*Women*<br>BMI ≤23 and GS ≤17<br>BMI 23.1–26 and GS ≤17.3<br>BMI 26.1-29 and GS ≤18<br>BMI >29 and GS ≤21 = 1<br>GS above cut-off within BMI strata = 0

20	Waist circumference (cm)	Men: ≥102 cm = 1, <102 cm = 0	Women: ≥88 cm = 1, <88 cm = 0
21	BMI outside normal (22–27 kg/m^2)	BMI <22 or >30 = 1, BMI 27–30 = 0.5, BMI 22–27 = 0	
	**Self-reported health and function**		
22	Own health in general	Very bad = 1, Bad = 0.75, Neutral = 0.5, Good = 0.25, Excellent = 0	
23	Own health compared with others of same age	Much worse = 1, A little worse = 0.75, About the same = 0.5, A little better = 0.25, Much better = 0	
24	Own dental health	Very bad = 1, Bad = 0.75, Neutral = 0.5, Good = 0.25, Excellent = 0	
25	Falls previous year	>1 fall = 1, 1 fall = 0.5, No falls = 0	
26	Unintentional weight loss in previous 6 months	Yes = 1, No = 0	
27	Mobility (walk about)	Unable to walk about = 1, Severe problems = 0.75, Moderate problems = 0.5, Slight problems = 0.25, No problems = 0	
28	Self-care (dress and wash)	Unable to dress and wash = 1, Severe problems = 0.75, Moderate problems = 0.5, Slight problems = 0.25, No problems = 0	
29	Usual activities	Unable to perform usual activities = 1, Severe problems = 0.75, Moderate problems = 0.5, Slight problems = 0.25, No problems = 0	
	**Motivation and attitudes**		
30	Depression	Very much = 1, Pretty much = 0.67, A little = 0.33, Not at all = 0	
31	Anxiety	Very much = 1, Pretty much = 0.67, A little = 0.33, Not at all = 0	
32	Feeling that everything is a struggle	Very much = 1, Pretty much = 0.67, A little = 0.33, Not at all = 0	
33	Not feeling happy	Not at all = 1, Not often = 0.67, Sometimes = 0.33, Most of the time = 0	
	**Vitality and life quality**		
34	Problems sleeping	Very much = 1, Pretty much = 0.67, A little = 0.33, Not at all = 0	
35	Life satisfaction	Not satisfied = 1, A little satisfied = 0.5, Very satisfied = 0	
36	Feeling hopeless about the future	Very hopeless = 1, Pretty hopeless = 0.67, A little hopeless = 0.33, Not hopeless = 0	
37	Loneliness	Not satisfied with number of good friends = 1, Partly satisfied = 0.5, Satisfied = 0	
38	Do not believe in self	Not at all = 1, Not much = 0.67, Sometimes = 0.33, Most of the time = 0	
	**Cognition and memory**		
39	MMSE score	≤10 = 1, 11–17 = 0.75, 18–20 = 0.5, 21–24 = 0.25, >24 = 0	
40	Impaired memory	Extreme problems = 1, Severe problems = 0.75, Moderate problems = 0.5, Slight problems = 0.25, No problems = 0	
41	Problem with daily tasks	Yes = 1, No = 0	

BMI, body mass index; GS, grip strength; MMSE, Mini-Mental State Examination; SPPB, Short Physical Performance Battery.

Supplementary Table S4 Association between dietary trajectories over 21 years and frailty index score in Tromsø7 in multiple imputation sample (*n* = 1998)

**Dietary trajectories**	**Model 1**		**Model 2**	
	β	95% CI	*β*	95% CI
Very unhealthy increase	-0.002	-0.02;0.01	-0.003	-0.02;0.01
Unhealthy	Ref.		Ref.	
Moderately healthy	-0.02	-0.03;-0.006	-0.01	-0.02;-0.002
Moderate increase	-0.02	-0.04;-0.007	-0.01	-0.03;-0.0004
Very healthy decrease	-0.03	-0.05;-0.02	-0.03	-0.04;-0.02

Model 1 adjusted for baseline sex and age.
Model 2 adjusted for baseline age, sex, body mass index, self-reported health, smoking, social support, and education.

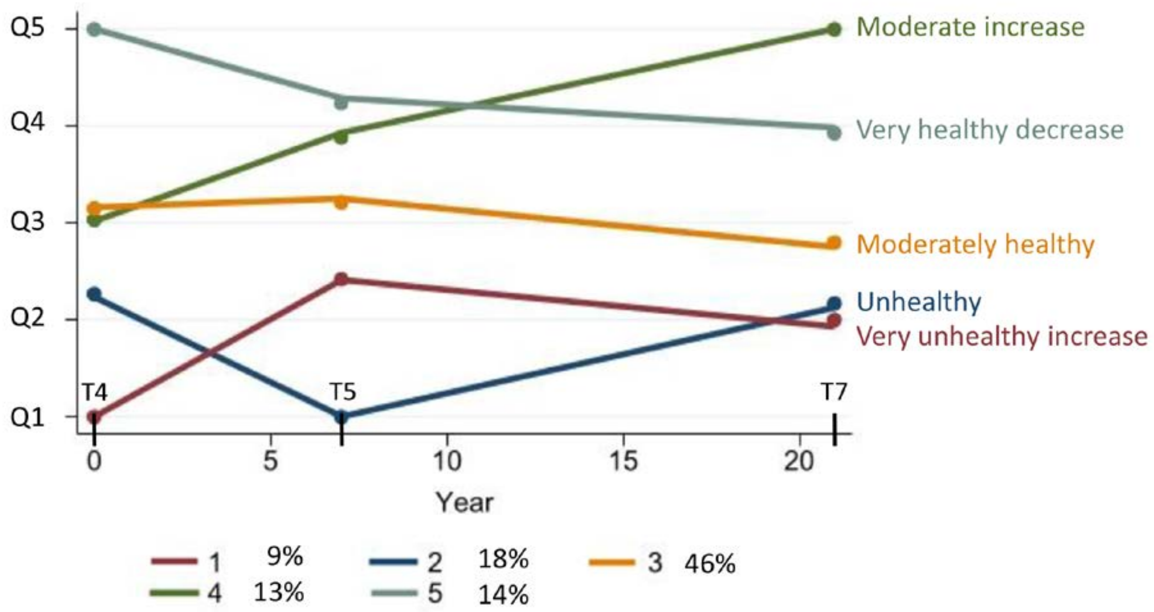

Supplementary Fig. S1 Dietary trajectories in MI sample (n=1998): very unhealthy increase (red, n=183), unhealthy (blue, n=366), moderately healthy (yellow, n=927), moderate increase (green, n=253), very healthy decrease (grey, n=269)**.**

Supplementary Table S5 Baseline characteristics and follow-up frailty status among participants included in multiple imputation (MI) analysis by level of missing food data (n = 1998)

**Baseline characteristics, Tromsø4**	Main sample (n = 715)[a]	Participants with >10% missing (n = 1283)[b]	p
Women, n (%)	391 (54.7)	764 (59.6)	0.04
Age, mean (SD)	53.9 (5.0)	55.7 (5.2)	<0.001
BMI, mean (SD)	25.4 (3.3)	25.9 (3.6)	0.003
Cohabitant, n (%)	614 (85.9)	1084 (84.5)	0.41
Social support[c], n (%)	561 (80.3)	1057 (86.4)	<0.001
Good health, n (%)	521 (73.0)	806 (62.9)	<0.001
Education[d], n (%)			
Primary	211 (29.6)	597 (46.7)	
Secondary	282 (39.6)	396 (31.0)	<0.001
Higher	220 (30.9)	285 (22.3)	
Smoking, n (%)			
Never	254 (35.6)	457 (35.7)	
Previously	252 (35.3)	490 (38.3)	0.25
Daily	208 (29.1)	333 (26.0)	
Inactivity, n (%)	212 (29.7)	464 (36.4)	0.003
Comorbidity[e], n (%)	4 (0.6)	27 (2.1)	0.007
**Follow-up, Tromsø7**			
Frailty index, mean (SD)	0.22 (0.09)	0.24 (0.09)	0.0003
Frailty[f], n (%)	234 (32.7)	520 (40.5)	0.001

P-values from t-test for continuous variables, Chi2 for categorical variables. [a]Main sample with ≥90% completed FFQ. Values in specific variables might vary slightly compared with Table 1, as MI will have been applied in variables with missing data. [b]Participants excluded from original analysis owing to <90% completed FFQ. Included in MI analysis if ≥25% completed relevant FFQ data. [c]Good social support defined as self-reported satisfied with number of good friends. [d]Education: primary (7–10 years), secondary (vocational middle school/senior high school/high school diploma), higher education (college/university). [e]Comorbidity: ≥2 of the following diseases (present or previous): diabetes, cancer, cardiovascular disease, chronic lung disease. [f]No test performed, owing to few observations (n<5) in cell. [g]Frailty defined as frailty index score ≥0.25.

Supplementary Table S6 Characteristics of participants in Tromsø4 by later participation status (n = 6949)[a]

**Characteristics in Tromsø4**	Re-attended Tromsø5 and Tromsø7 (n = 1544)	Dropped out after Tromsø4 and did not re-attend (n = 1845)	p^b
Women, n (%)	885 (57.3)	770 (41.7)	<0.001
Age (years), mean (SD)	54.9 (5.3)	52.1 (7.1)	<0.001
BMI (kg/m^2), mean (SD)	25.6 (3.4)	25.7 (4.0)	<0.001
Cohabitant, n (%)	1311 (84.9)	1352 (73.4)	<0.001
Social support[c], n (%)	1245 (82.9)	1410 (78.9)	0.002
Good health, n (%)	1045 (67.7)	1168 (63.5)	<0.001
Education[d], n (%)			
Primary	574 (37.3)	649 (35.3)	
Secondary	557 (36.2)	630 (34.2)	<0.001
Higher	409 (26.6)	561 (30.5)	
Smoking, n (%)			
Never	545 (35.3)	454 (24.6)	
Previously	565 (36.6)	540 (29.3)	<0.001
Daily	433 (28.1)	851 (46.1)	
Inactivity, n (%)	518 (33.7)	735 (40.0)	<0.001
Comorbidity[e], n (%)	15 (1.0)	61 (3.3)	<0.001

[a]Participants in Tromsø4 ≥44 years with >90% completed FFQ and no extreme energy intakes (<1 and >99 percentiles). [b]P-values: test for differences in Tromsø4 characteristics between participants with different levels of later participation. T-test for continuous variables, chi2 for categorical variables. [c]Good social support defined as self-reported satisfied with number of good friends. [d]Education: primary (7–10 years), secondary (vocational middle school/senior high school/high school diploma), higher education (college/university). [e]Comorbidity: ≥2 of the following diseases (present or previous): diabetes, cancer, cardiovascular disease, chronic lung disease.
